# Research on the state of blended learning among college students – A mixed-method approach

**DOI:** 10.3389/fpsyg.2022.1054137

**Published:** 2022-12-01

**Authors:** Chao Deng, Jiao Peng, ShuFei Li

**Affiliations:** ^1^Information Management and Information System, Guangdong University of Science and Technology, Dongguan, China; ^2^School of Artificial Intelligence, Dongguan Polytechnic, Dongguan, China

**Keywords:** blended learning, TAM 3, learner engagement theory, technology acceptance, student engagement

## Abstract

In the wake of the COVID-19 pandemic in 2019, China’s education leaders began to focus on and promote blended learning. The process is still in its infancy in Chinese colleges and universities, and its development remains a problem to be solved. By combining technology acceptance and student participation, this article proposes an analysis model for assessing the factors influencing blended learning. A questionnaire was designed and distributed, and 796 valid responses were collected. The mean and variance were used to examine the status of students’ technology acceptance and satisfaction with blended learning. The *t*-test method was employed to analyze the gender differences between students in regard to the topic. The results show that: (1) students majoring in computer science view the factors as having a high level of influence in blended learning. (2) There are major variances regarding the perception of service quality between male and female computer science major students. There is no significant difference between them in terms of perceived usefulness, perceived ease of use, or computer self-efficacy. (3) There are considerable disparities in the skill involvement and participation of computer science major college students. The results show that the technology acceptance and participation of students determine the effect of blended learning. Based on these findings, this article provides theoretical and practical suggestions for the implementation of blended learning to improve its effect.

## Introduction

COVID-19 emerged suddenly in December 2019. This virus is highly infectious and difficult to control. To inhibit the spread of the epidemic, the Chinese government resolutely implemented stringent prevention and control measures, and schools stopped normal classroom teaching activities. E-learning became the only choice of teaching methods at that time. The epidemic situation promoted the comprehensive popularization of e-learning, as well as people’s thinking about blended learning. The motivation of this research is with the fast advancement of computer information technology represented by 5G and big data, the application of new technology has enriched teachers’ online teaching methods and the effectiveness of students’ learning, and the essential requirements for the rapid development of blended learning are also in place.

Lin and Xie (2019) argue that the zero-lag and high-speed characteristics of 5G communication technology have changed the situation regarding learning in fixed locations, and mobile learning has also become popular. Wang (2017) believes that big data technology digitizes every student’s learning behavior. Through data analysis, students’ learning status can be understood, after which teaching objectives can be made more specific, accurate and personalized. Qi and Chen (2022) state that through the analysis of students’ data, the correlation and matching between their learning needs and status have been improved, and the effectiveness of online teaching has been enhanced.

Since 2016, more enterprises have chosen the blended learning mode for staff training, and an increasing number of schools regard it as one of the important ways of carrying out teaching activities. Based on the above policy background and the current state of information technology, blended learning will become one of the important elements of China’s higher education reform (Ministry of Education, 2021).

At the present stage, education, training, and other fields have fully recognized the huge advantages of blended learning. However, these are not inherent. Teachers and managers must not only carefully plan learning activities and choose appropriate learning media, but also add new ideas to make up for the deficiencies, expand the advantages and enhance the teaching effect.

In the context of its gradual promotion, the problem this research seeks to address is how to better develop blended learning. This study takes the factors that influence blended learning as the starting point and suggests a hypothesis based on a consideration of the literature as well as existing relevant theories [including the technology acceptance model 3 (TAM 3)]. A questionnaire is used as a research tool to examine the acceptability and psychological factors of teachers and students.

To sum up, the research issues of this study are:

(1) The extent of the factors influencing blended learning in computer science courses in terms of perceived usefulness and ease of use, computer self-efficacy and service quality.
(2) Whether there are significant differences in the factors influencing blended learning in terms of gender when the respondents are grouped according to their profile variables.
(3) The level of student engagement in blended learning in terms of skills, emotional, and performance engagement, as well as participation/interaction.
(4) Whether there are significant gender differences in student engagement in blended learning.
(5) Whether the factors influencing blended learning are significantly related to student engagement.

## Literature review and hypotheses

Blended learning has been a research hotspot in relation to international educational technology in recent years. Many scholars believe that blended learning is mainly a combination of offline and online activities. According to Aycock et al. (2002), blended learning combines the advantages of face-to-face and online learning in a balanced way, so that they can be maximized. Michael and Heather (2015), Huang et al. (2020), and Ma (2020) all argue that blended learning means that part of the teaching time is used for face-to-face teaching in school, and the rest for the online type. Online teaching can flexibly control the time, place and method of instruction.

### Factors influencing blended learning

Blended learning is not an independent process. Before this analysis, many scholars examined the factors involved in blended learning from the perspective of technology acceptance, as well as using the TAM, which provides support for this study.

The factors influencing blended learning can be subdivided into the following aspects from the perspective of technology acceptance: perceived ease of use and usefulness, computer self-efficacy, service quality, subjective norms, etc.

Scholars have examined the influence of different factors on students’ willingness to accept blended learning, as well as their learning participation, behavior, and effect. Lv and Ge (2020), through the integration of technology acceptance and planned behavior theories, studied the acceptance willingness of college students in hybrid teaching in five aspects: perceived usefulness and ease of use, use attitude, subjective norms, and perceived behavior control, exploring measures and countermeasures to enhance the willingness of college students to accept the blended learning model from the perspective of students’ subjective psychology.

### Perceived ease of use

Lv and Ge (2020) and Wu et al. (2020) pointed out that the perceived ease of use is one of the important prerequisites for determining perceived usefulness. In blended learning, the stronger the perceived ease of use of blended learning courses, the more willing students are to actively invest in blended learning and the easier it is to feel its effect. Zhang et al. (2016) and Liu and Zhang (2020) proved through empirical evidence that perceived ease of use is a key factor that affects users’ willingness to use. Based on the above analysis, the first assumption proposed in this article is:

**H1:** Perceived ease of use has a positive impact on students’ acceptance of technology in blended learning.

### Perceived usefulness

Perceived usefulness means that students feel the effectiveness of blended learning in improving the learning effect. Han (2012) believed that personal factors affect the acceptance of technology through perceived ease of use or usefulness. In addition, Li (2019) proved through experiments that when college students perceive mobile learning as useful, their behavioral willingness to carry it out is stronger. Wang and Huang (2020) pointed out that perceived usefulness has a positive impact on students’ online active learning intention. Through practical research, Qin et al. (2021) and Jing et al. (2021) have both evidenced that the most influential factor in students’ willingness to use online teaching is perceived usefulness. Based on the above analysis, one assumption proposed in this article is:

**H2:** Perceived usefulness has a positive impact on students’ acceptance of technology in blended learning.

### Computer self-efficacy

Self-efficacy is a concept proposed by Bandura (1977), who believed that people’s judgment of their own abilities plays an important role in people’s self-regulation system, and thus suggested the concept of self-efficacy. Through experimental research, it has been found that the application of the network learning space can gradually improve the Internet self-efficacy of college students, and the cumulative application effect is very significant (Xie et al., 2016). The Internet self-efficacy of students in the experimental group using smart classrooms such as Peng was significantly higher than that of the control group students utilizing traditional multimedia ones (Ke et al., 2019; Ke and Zhang, 2020; Li X. et al., 2020; Li Y. Y. et al., 2020; Li X. Y. et al., 2022). Improving students’ perception of the environment of the smart classroom is very significant for enhancing their self-efficacy (Peng et al., 2021).

Self-efficacy affects students’ willingness to engage in blended learning and the learning effect. The more confident students are and the stronger their control over external conditions, the more willing they are to accept blended learning (Prasad et al., 2018). Zhou and Lou (2021) demonstrated that the online learning self-efficacy of college students positively predicts their deep learning level. Chang et al. (2014) found that self-efficacy also has a certain impact on learning motivation, as well as the process and results. Meanwhile, Yerdelen-Damar and Peşman (2013) posited that self-efficacy can explain the differences in physical behavior between different genders.

Liu (2013) pointed out that the online learning environment is highly correlated with computer self-efficacy, as well as that optimizing the online learning environment has practical significance for improving the computer efficiency of college students. Liu proposed verbal encouragement and support for learners’ computer use ability, in addition to awakening improving strategies for low-efficiency people with stress and anxiety. In addition, Fang (2015) and Zhao et al. (2015) both argued in their respective studies that self-efficacy can affect the perceived ease of use, which in turn affects the individual’s technical acceptance. Li Y. Y. et al. (2020) proved through empirical research that self-efficacy affects students’ satisfaction with online learning. Based on the above analysis, another assumption proposed in this article is:

**H3:** Self-efficacy has a positive effect on students’ technical acceptance.

### Service quality

Service quality refers to the degree of adaptation between the user’s task requirements and personal capabilities, as well as the technical support provided by the system. Service quality affects the intention to use blended learning through perceived usefulness. Xie and Zhu (2012) regarded effective teaching content and the richness of course content as sub-items under the teaching support system dimension. Zhang and Li (2014) confirmed that when teachers use a network platform to carry out teaching, they also need to pay attention to the needs of the teaching task. Zhou et al. (2009) pointed out that when users accept a new technology, it is often because it meets their needs better than the old one, that is, the new technology better matches the task. Based on the above analysis, an assumption proposed in this article is:

**H4:** Service quality influences students’ technical acceptance of blended learning.

### Technology acceptance model 3

The TAM specifically deals with the prediction of the acceptability of the information system. The TAM is a technical one that is widely employed by researchers in studying the factors influencing blended learning. It is a relatively simple and comprehensive research model compared to others. Its purpose is to predict the acceptability of the tool and determine what changes must be made to the system to make it acceptable to users. This model shows that the acceptability of an information system is determined by two main factors: perceived usefulness and ease of use (Fu et al., 2020, 2021a,2021b). In TAM 3, these aspects are determined by four different types of factors, including individual differences, system characteristics, convenience conditions and community influence.

Zhang and An (2015) improved the original TAM and created an online course acceptance model with seven latent variables that represented the characteristics of students’ online learning. These included the two variables of perceived usefulness and ease of use from the perspective of students, as well as content, function, attractive design, and brand image, which were employed as the four variables of the online course. The intention of students’ use behavior was also set as a variable. It was concluded that perceived usefulness, course content, brand image, function, and attractiveness have a positive effect on college students’ acceptance of online courses, and perceived ease of use has a positive effect on college students’ acceptance of online courses (Liu G. et al., 2020; Liu N. et al., 2020; Liu et al., 2021, 2022a,b).

Likewise, Gao and Gao (2011) believed that individual differences refer to the individual’s perception of the impact of personality characteristics on perceived usefulness and ease of use. System characteristics refer to the individual’s perception of the usefulness and ease of use of the system. Community influence refers to the influence of individuals on society. The process and mechanism of information technology ability perception encourage individuals to form such an impression, and the convenience condition is that society supports individuals in using information technology.

Wu (2014) stated that compared with previous models, TAM 3 is more comprehensive and has a wider scope of application. It has an irreplaceable advantage in enhancing users’ technical acceptance and management time. Fang (2015) applied the TAM 3 model to study the factors affecting the learning behavior of students using massive open online courses (MOOCs) and concluded that there is a significant positive correlation between learners’ MOOC learning intentions and behaviors; perceived usefulness is significantly positively correlated with learning intention. Perceived ease of use is also positively correlated with learning intention, but not significantly; the biggest influence on perceived ease of use is the students’ sense of computer self-efficacy.

### Gender differences

Gender differences represent an area of psychological research, and related topics still attract the attention of scholars. Researchers posited that studies on gender differences have important theoretical value and practical significance for the different cognitive and behavioral traits of men and women in real life. In China, people place different expectations on individuals of different genders during the process of growing up, which may lead to deviations in the acceptance of new things by college students of different genders. Therefore, gender differences form a part of this research.

Ongowo and Hung (2014) claimed that girls’ self-regulation ability is greater than that of boys. In addition, Zhang and Li (2018) believed that the self-regulation ability of female college students in the online learning environment is significantly higher than that of male students. Likewise, Zhu et al. (2012) argued that with the development of learning, female learners show a strong ability to deal with learning difficulties and regulate emotions, while male ones demonstrate the opposite.

Song (2014) found that there are gender differences in internal goals, as well as learning plans, control and management, in addition to self-efficacy between male and female college students through an empirical study of the online autonomous learning of non-English majors. Female learners demonstrate a stronger motivation to learn. Li (2016) also came to a similar conclusion. Al-Azawei (2019) concluded that gender differences had only a slight moderating effect on the relationship between e-learning self-efficacy and learning management system (LMS) acceptance. Self-efficacy had a stronger impact on the intention to use LMSs among men than among women.

On another note, Punnoose (2012) stated that men’s willingness and behavior toward learning online are higher than that of women. In terms of willingness to learn, men and women show higher interest and more open attitudes than women, and express a stronger willingness to use in the future (Punnoose, 2012). Antonio and Tuffley (2014) believed that women’s perceived and actual use of the Internet is less profitable than men, which leads to women’s inactive attitudes toward online activities. Based on the above analysis, the assumptions proposed in this article are:

**H5:** There is no significant difference between male and female computer science major college students in perceived usefulness.

**H6:** There is no significant difference between male and female computer science major college students in perceived ease of use.

**H7:** There is no significant difference between male and female computer science major college students in computer self-efficacy.

**H8:** There is a significant difference between male and female computer science major college students in their perception of service quality.

**H9:** There is a significant difference between male and female computer science major college students in terms of engagement.

### Learner engagement

Learner engagement refers to how actively involved and immersed learners are in the course they are taking. In this study, the researchers believe that for learning to be successful, everyone involved must be engaged. The quality of engagement can directly affect the final learning effect.

Halverson and Graham (2019) added that personal and contextual facilitators of engagement, including learner characteristics and thoughtful learning experience design, can increase the likelihood of learner engagement, and learner engagement in blended learning environments will support advances in blended learning engagement research that is increasingly real-time, minimally intrusive, and maximally generalizable across subject matter contexts (Cheng et al., 2018a,b, 2021a,2021b; Cheng R. et al., 2019; Cheng Y. et al., 2019; Chen et al., 2020, 2021; Dai et al., 2020).

Learner engagement refers to the effort the learner makes in promoting his or her psychological commitment to stay engaged in the process of learning to acquire knowledge and build his or her critical thinking (Dixson, 2015). Richards (2011) stressed that meaningful learning occurs when learners are actively engaged. There are different models for measuring learner engagement in different contexts.

Dixson (2015) validated the Online Student Engagement (OSE) scale model using the concept of behavioral engagement, which consists of what was earlier described as observational and application learning behaviors. Zhang and Wang (2021) believed that student engagement directly or indirectly affects the growth and development of students in all aspects of the university. Rajabalee et al. (2020) established that student engagement is an important factor that contributes to the success of students on online courses. Engagement should be explained from three aspects: behavioral, emotional, and cognitive engagement. Based on the above analysis, the final assumption proposed in this article is:

**H10:** Engagement has a positive impact on students’ acceptance of technology in blended learning.

## Conceptual model

**FIGURE 1:**
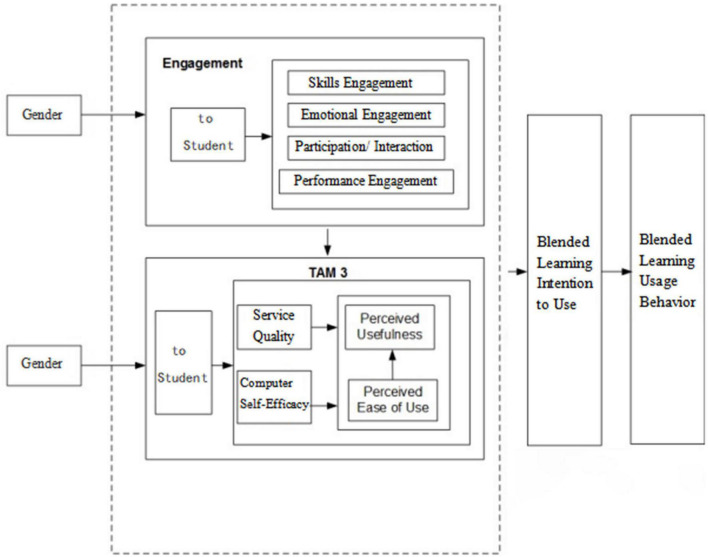
Conceptual model.

On the basis of the TAM 3 model, this study designed a new research model using the two aspects of technology acceptance and learner participation (see Figure 1) to examine the factors influencing blended learning and analyze its impact from the perspectives of technology acceptance and student participation.

## Research methods

This research chiefly reviewed the literature from four aspects, including the factors influencing blended learning, TAM 3, learner engagement and gender differences. This section mainly used the qualitative research method.

The Statistical Package for the Social Sciences (SPSS) software then was used to analyze the numerical data, mainly including the validity and reliability analyses of the questionnaire, as well as the factors influencing blended learning and the differences based on the profile variables of the respondents.

The research took place in Dongguan, Guangzhou, Yang Jiang, and Foshan in Guangdong Province. The random sampling method was adopted to select public undergraduate universities and junior colleges, as well as private undergraduate universities and independent colleges, according to the different characteristics of the overall survey. The student sample size calculated by Slovin’s formula was 795, and the sample size of each school was allocated to the proportion of the total number of students. Details of the respondents are provided in Table A in Supplementary Appendix A.

The researcher sought permission and assistance from the relevant authorities conducting the survey. Based on the purpose of the research, the researchers designed and developed a questionnaire named “Factors influencing blended learning for students” to collect the data.

The researcher introduced the purpose of the study and the content of the questionnaire to school administrators by phone or email, and then applied to distribute the questionnaire in the school. The questionnaire on the factors influencing blended learning for students was administered to computer science major students through Questionnaire Star. After the application was approved, the researcher was allowed to circulate the questionnaire. The researcher then went to the school and gave the questionnaires to the respondents.

Based on the data analysis results of this study, the researcher identified the relationships between the factors influencing blended learning, as well as reflecting on the research conclusions in addition to the actual situation and real problems concerning blended learning.

## Instruments

Only one data gathering instrument was used in this study, which was the survey questionnaire (see Table A in Supplementary Appendix B). This was developed by the researcher, then validated by the adviser as well as selected experts in the fields of (blended learning) education and evaluation studies. To improve the reliability of the formal survey, a preliminary survey was conducted first. A total of 30 pre-questionnaires were sent over the Internet. In the preliminary investigation, the overall Cronbach’s α coefficient was 0.980, and the Cronbach’s α coefficient of each indicator ranged from 0.848 to 0.936. This indicated that the reliability of the factors was acceptable.

## Participants

The survey was conducted through an online questionnaire among students from nine universities in Guangdong. The invited students could choose whether or not to participate in the survey as they wished and were informed of the purpose of the survey. Online questionnaires were issued and collected for a month. After data cleaning, 796 valid questionnaire responses were obtained.

## Results

In this study, the mean and standard deviation were utilized to analyze the factors affecting blended learning (see Table 1) and the current situation of students’ input level in blended learning for a computer science major (see Table 2). Students majoring in computer science were divided into groups according to gender, and a *t*-test was employed to analyze the gender differences in the factors influencing mixed learning among computer science students (see Table 3) and the variations in the degree of students’ participation in mixed learning (see Table 4). The Pearson correlation was used to analyze the correlation between the factors influencing blended learning and the level of student involvement (see Table 5).

**TABLE 1:** Level of the factors influencing blended learning at a computer science college.

Indicators		Mean	Viewpoint	Ranking	SD
Student	Perceived usefulness	3.546	H	1	0.9908
	Perceived ease of use	3.446	H	3	1.0026
	Computer self-efficacy	3.472	H	2	0.9745
	Service quality	3.403	H	4	1.0071
	Composite mean	3.467	H		0.994

Indicators: Influencing factors of students’ blended learning Level.

Mean: Average score of each indicator given by respondents.

Viewpoint: The grade of the Mean.

Ranking: Ranking of Mean of this indicator.

SD: The standard deviation of the respondents’ rating of this indicator.

1.00–1.80 very low (VL), 1.81–2.60 low (L), 2.61–3.40 average (A), 3.41–4.20 high (H), and 4.21–5.00 very high (VH).

**TABLE 2:** Level of engagement of computer science college students.

Indicators		Mean	Viewpoint	Ranking	SD
Student	Skills engagement	3.381	A	1	0.994
	Emotional engagement	3.349	A	4	1.016
	Participation/interaction	3.361	A	3	1.001
	Performance engagement	3.369	A	2	1.004
	Composite mean	3.365			0.013

1.00–1.80 very low (VL), 1.81–2.60 low (L), 2.61–3.40 average (A), 3.41–4.20 high (H), and 4.21–5.00 very high (VH).

**TABLE 3:** Differences in the factors influencing the blended learning of computer science college students in terms of gender.

Indicators	Gender	Mean	*t*-Test for equality of means				
			*t*-Value	Df	*P*-value	Decision	Interpretation
Perceived usefulness	Male	3.52	−0.895	794	0.371	Accept Ho	No significant difference
	Female	3.585					
Perceived ease of use	Male	3.393	−1.79	794	0.074	Accept Ho	No significant difference
	Female	3.523					
Computer self-efficacy	Male	3.43	−1.452	794	0.147	Accept Ho	No significant difference
	Female	3.533					
Service quality	Male	3.324	−2.716	794	0.007**	Reject Ho	Significant difference
	Female	3.52					

**The correlation is strongly significant at the 0.01 level (two-tailed). H0 means accepting the assumption.

**TABLE 4:** Differences in the engagement of computer science college students in terms of gender.

Indicators	Gender	Mean	*t*-Test for equality of means				
			*t*-Value	Df	*P*-value	Decision	Interpretation
Skills engagement	Male	3.297	−2.934	794	0.003**	Reject Ho	Significant difference
	Female	3.506					
Emotional engagement	Male	3.258	−3.085	794	0.002**	Reject Ho	Significant difference
	Female	3.483					
Participation/interaction	Male	3.276	−2.906	794	0.004**	Reject Ho	Significant difference
	Female	3.486					
Performance engagement	Male	3.291	−2.653	794	0.008**	Reject Ho	Significant difference
	Female	3.482					

**The correlation is strongly significant at the 0.01 level (two-tailed). H0 means accepting the assumption.

**TABLE 5:** Pearson correlation analysis between the factors influencing blended learning and level of student engagement.

		Perceived usefulness	Perceived ease of use	Computer self-efficacy	Service quality	Engagement
Perceived usefulness	Pearson correlation	1				0.745**
	Significant (two-tailed)					0
	*N*	796				796
Perceived ease of use	Pearson correlation		1			0.896**
	Significant (two –tailed)					0
	*N*		796			796
Computer self-efficacy	Pearson correlation			1		0.895**
	Significant (two –tailed)					0
	*N*			796		796
Service quality	Pearson correlation				1	0.924**
	Significant (two-tailed)					0
	*N*				796	796
Engagement	Pearson correlation	0.745**	0.896**	0.895**	0.924**	1
	Significant (two-tailed)	0	0	0	0	
	*N*	796	796	796	796	796

**The correlation is significant at the 0.05 level (two-tailed).

Table 1 shows that the mean values of perceived usefulness and ease of use as well as computer self-efficacy have all reached “H,” so they all have a positive impact on students’ acceptance of technology in blended learning. H1, H2, and H3 are therefore valid.

The mean value of service quality is “H,” which proves that it has a positive impact on students’ acceptance of technology in blended learning. H4 is also valid.

Table 3 shows that the *P*-value of perceived usefulness, perceived ease of use and computer self-efficacy is greater than 0.05, indicating that there is no significant difference. Therefore, H5, H6, and H7 are valid. The *P*-value of service quality is less than 0.01, indicating a significant difference between male and female computer science major college students in their perception of service quality. H8 is also valid.

Table 4 demonstrates that the *P*-values for skills, emotional, and performance engagement, as well as participation/interaction, are all less than 0.01, indicating that there was a significant difference between male and female computer science major college students in terms of engagement. H9 is therefore valid.

As shown in Table 5, the correlations between perceived ease of use, perceived usefulness, self-efficacy, and engagement are significant. The Pearson correlation relating to service quality reached 0.924, and its correlation with engagement is also significant. This means that service quality has a positive impact on students’ acceptance of technology in blended learning. Therefore, H10 is valid.

This is consistent with the results of a recent study which found that to develop new skills and acquire new knowledge, individuals must consciously mobilize and devote some of their physical and psychological (cognitive and emotional) energy to it; emotional and cognitive engagement are the most fundamental expressions of learner engagement (Janosz, 2012). Pekrun (2011) argued that emotions influence a broad variety of cognitive processes that contribute to learning, such as perception, attention, memory, decision-making, and cognitive problem-solving. Situational interest, or enjoyment created by external stimuli, is a temporary affective state that indicates emotional energy expended and created by learning efforts. Although short-lived, this interest focuses attention, enhances cognitive performance and learning, and improves integration.

## Discussion

### Levels of the factors influencing the blended learning of computer science major students

Four factors influence blended learning: perceived usefulness, perceived ease of use, computer self-efficacy, and service quality.

In Table 3, the composite mean of the factors influencing blended learning among students is 3.467, reflecting a high level, which is a moderately preferred value, indicating that computer science students believe that blended learning is useful for learning and is relatively easy to use. At the same time, they observe that their ability to learn from as well as the service quality of the blended learning platform are also good.

The factors influencing blended learning have been derived according to the TAM theory, and the TAM deals more specifically with the prediction of the acceptability of the information system. The purpose of this model is to predict the acceptability of the tool and determine what changes must be made to the system for it to be acceptable to users. This model shows that the acceptability of an information system is determined by two main factors: perceived usefulness and perceived ease of use. Zhang and Li (2014) used a comprehensive version of the TAM to successfully carry out research on blended learning behaviors in colleges and universities, and found that perceived usefulness, perceived ease of use, and self-efficacy. The practicality of the system also has a positive effect on intention in terms of blended learning behavior.

Perceived usefulness reflects the degree to which a person believes that the use of a specific system will improve their work performance. In Table 3, the student’s perceived usefulness composite mean value is 3.5465 or a high level, which means that computer science students believe that blended learning is useful for learning, and this value is good, higher than the average level. The mean value of perceived usefulness implies it is useful to develop blended learning among computer science students, and that blended learning should be considered in more subjects at a later stage.

### Significant differences in the factors influencing blended learning when grouped according to gender

Based on the data analysis, there is no significant difference in perceived ease of use, perceived usefulness or computer self-efficacy between male and female computer science students. There are however major variations in the perception of service quality between male and female computer science major college students. Gender has a significant moderating effect on service quality. Girls are more aware than boys of a blended learning platform in terms of the degree to which learning objectives and teaching activities need to be matched. Xiong (2015) studied the factors influencing college students’ acceptance of mobile learning, and believed that gender plays a regulating role in students’ acceptance of mobile learning. This is consistent with the conclusion of this study.

### Level of engagement of computer science major college students in blended learning

Engagement refers to the time and energy that students spend in and out of the classroom and on effective educational activities, as well as the services and conditions created by universities to promote students’ participation in these educational activities. In this study, engagement refers to the students’ motor skills and internal investment in the blended learning process.

According to the analysis results, the computer science major college students’ composite mean value for engagement is 3.365, with the mean values all between 3.349 and 3.381, reflecting that it is an “average level.” The score for skills engagement is highest, with a composite mean value of 3.381. The score for emotional engagement is relatively low, with a score of 3.349.

In Table 2, the mean value of engagement is 3.365 or an average level, which can verify the promotion of participation in technology acceptance. The higher the level of student engagement, the better their academic performance. Zhu (2010) likewise stated that student participation in learning has the most significant positive impact on student growth. Whether it is knowledge accumulation, cognition, thinking, ability improvement, or value establishment, the positive effect of student participation is significantly higher than other research factors.

### Significant differences in computer science major college students’ degree of engagement

The results reveal that there are significant differences in “skills engagement,” “emotional engagement,” “participation/interaction,” and “performance engagement” among male and female computer science major college students. Gender has a significant moderating effect on skills, emotional, and performance engagement, as well as participation/interaction. In the four participation factor sub-items, the mean value for girls is higher than that for boys. This shows that the internal and external investment of girls in blended learning is significantly higher than that of boys. A recent paper found the emotional engagement level of girls in blended learning is significantly higher than that of boys, which is consistent with the conclusions of this study (Ge, 2019). Zhu et al. (2012) claimed that with the development of learning, female learners demonstrate a strong ability to deal with learning difficulties and regulate emotions, while male learners are the opposite. There are significant gender differences in the use of learning strategies among college students. In autonomous learning, the proportion of girls using learning strategies, especially the metacognitive and affective types, is higher than that of boys (Zhang, 2021).

### Correlation analysis of the factors influencing blended learning

Table 5 shows that when the selected level is 0.05, there is a positive correlation between the factors influencing blended learning and student engagement, which is embodied in perceived usefulness, perceived ease of use, computer self-efficacy, and service quality. A recent study found that to develop new skills and acquire new knowledge, individuals must consciously mobilize and devote some of their physical and psychological (cognitive or emotional) energy to the task; emotional and cognitive engagement are the most fundamental expressions of learner engagement (Janosz, 2012). Pekrun (2011) argued that emotions influence a broad variety of cognitive processes that contribute to learning, such as perception, attention, memory, decision-making, and cognitive problem-solving. Situational interest, or enjoyment created by external stimuli, is a short-lived affective state that indicates emotional energy expended and created by learning efforts. Although temporary, this interest focuses attention, enhances cognitive performance and learning, and improves integration.

## Conclusion

This research explored the factors influencing blended learning for computer science students, as well as how to strengthen it. The researcher conducted the study by examining the literature, as well as through questionnaire surveys and data analysis, and proposed training programs that enhance blended learning to improve the overall level for computer teachers and students in colleges and universities. The conclusions of this evaluation are as follows:

(1) The computer science students perceived the factors influencing blended learning as having a relatively “high level.” The students scored perceived usefulness highest and quality service lowest.
(2) There is a significant difference between female and male computer science students in perceptions of service quality. There is no major variation between them in terms of perceived usefulness, perceived ease of use and computer self-efficacy.
(3) The computer science major students have an “average level” of engagement. The students scored highest in skills engagement and lowest in emotional engagement.
(4) There is a significant difference between male and female computer science major college students in terms of skills, emotional, and performance engagement, as well as participation/interaction.
(5) There are significant differences between the factors influencing blended learning and the level of engagement.
(6) There is a significant difference between female and male computer science major students in terms of engagement.

### Limitations

The factors influencing blended learning involve many aspects, such as the degree of computer informatization, as well as economic conditions, internal factors and so on. In this study, the researcher conducted research based on the two aspects of technology acceptance and engagement, without considering external environmental factors.

This study used a quantitative analysis method, but due to the impact of the COVID-19 epidemic, travel has been restricted in China, so it was impossible to understand the psychological dynamics of the subjects by involving a greater number of research participants.

This analysis was restricted to the academic year 2020–2021. The survey respondents were limited to 2018 and 2019 students majoring in computer science, as well as computer teachers. The schools of the research participants were all in economically developed regions of China, and had the basic requirements for carrying out blended learning. The students and teachers had utilized blended learning since the epidemic, and before that, they were using offline classroom teaching.

### Recommendations

Based on the results of this study, the following suggestions are put forward to improve blended learning.

(1) To universities: strengthen the promotion of blended learning. Carry out promotional work in schools to increase its understanding among college teachers and students, enhance their sense of identity with blended learning, and attract more teachers and students to participate in it. With the addition of more high-quality enterprises for carrying out school-enterprise cooperation, in the holidays teachers and students should participate in enterprise-related work to provide the conditions for blended learning.
(2) To computer science major college students: students are advised to develop their basic computer and information technology training, improve their ability to use information technology and enhance their learning planning, as well as create clear learning objectives, make reasonable and feasible learning plans, refine their career planning and goals and participate in more group activities to improve their communication skills.
(3) To information platform companies: information platform companies design, optimize, and develop blended learning platforms based on the needs and usage habits of college teachers and students to meet the needs of teaching and learning. They may want to improve the operating convenience and user-friendliness of blended learning platforms and provide related service support (such as online training and problem-solving, etc.), so that teachers and students can fully explore the platforms’ functions to complete blended learning tasks, and attain a sense of accomplishment.
  1. To the training organizer: plan the course system well. In the process of training, the training organizers need to deal with the relationship between school curriculum and training content, to avoid repetition. For example, if the training content is similar to the existing curriculum content of schools, it should be integrated into the school teaching content, and the courses should be taught by the enterprise teachers together with schoolteachers, expanding the training mode. The establishment of multi-direction and multi-type training modes is beneficial for improving students’ comprehensive application ability.
  2. Further research: this study provides new views on the investigation methods and research conclusions adopted for the factors influencing blended learning which form the basis for its enhancement. This project considered college teachers and students majoring in computer science. In future research, blended learning could be studied in different types of schools and corresponding training plans could be specified.

## Data availability statement

The original contributions presented in this study are included in the article/Supplementary material, further inquiries can be directed to the corresponding author.

## Author contributions

CD and JP: conceptualization. CD: methodology and software, validation, investigation, resources, funding acquisition, supervision, and writing—original draft preparation, review, and editing. All authors read and agreed to the published version of the manuscript.
