# Supplementary material for: Research on the state of blended learning among college students – A mixed-method approach
Source: Front Psychol. 2022 Dec 1;13:1054137. doi: 10.3389/fpsyg.2022.1054137 (PMC9751935; doi:10.3389/fpsyg.2022.1054137)
Supplement: Supplementary file 2 [file Table_1.docx]

**Appendix A**

Table A Distribution of the Respondents

| **University** | **Teachers** | | **Students** | |
| --- | --- | --- | --- | --- |
|  | **Total number** | **Sample** | **Total Number** | **Sample** |
| **Public** |  |  |  |  |
| Guangdong University of Technology | 80 | 39 | 1600 | 137 |
| Dongguan Polytechnic | 36 | 17 | 640 | 55 |
| Foshan Polytechnic | 26 | 13 | 480 | 41 |
| Dongguan University of Technology | 22 | 11 | 400 | 34 |
| Guangdong Polytechnic of Science and Trade | 28 | 14 | 600 | 52 |
| YangJiang Polytechnic | 45 | 22 | 1160 | 100 |
| **Private** |  |  |  |  |
| City College of Dongguan University of Technology | 45 | 22 | 920 | 80 |
| Guangdong University of Science and Technology | 90 | 44 | 2400 | 206 |
| Guangdong Polytechnic College | 40 | 20 | 1060 | 91 |
| **Total** | **412** | **202** | **9260** | **796** |
