# Supplementary material for: Research on the state of blended learning among college students – A mixed-method approach
Source: Front Psychol. 2022 Dec 1;13:1054137. doi: 10.3389/fpsyg.2022.1054137 (PMC9751935; doi:10.3389/fpsyg.2022.1054137)
Supplement: Supplementary file 3 [file Table_2.docx]

**Appendix B**

Table A Factors Influencing Blended Learning for Students

**Part I. PROFILE OF THE RESPONDENTS**

**A．Gender**

**___ Male**

**___ Female**

**B. Year Level**

**___ First Year**

**___ Second Year**

**___ Third Year**

**___ Fourth Year**

**Part II. FACTORS INFLUENCING BLENDED LEARNING**

*Using the scale below, place a check (/) in the appropriate column for your response.*

*Legend:*

*5 – Strongly agree*

*4 – Agree*

*3 – Neither agree nor disagree*

*2 – Disagree*

*1 – Strongly disagree*

| **Perceived Usefulness** | **5** | **4** | **3** | **2** | **1** |
| --- | --- | --- | --- | --- | --- |
| 1. Blended learning can improve my learning efficiency. |  |  |  |  |  |
| 2. Blended learning can improve my academic performance. |  |  |  |  |  |
| 3. Blended learning can improve my self-learning ability. |  |  |  |  |  |
| 4. Blended learning can improve my professional development. |  |  |  |  |  |
| 5. Blended learning can improve my creative ability. |  |  |  |  |  |
| **Perceived Ease of Use** | **5** | **4** | **3** | **2** | **1** |
| 1. It is easy to operate the blended learning platform (ChaoXing learning system). |  |  |  |  |  |
| 2. It is easy to communicate and interact with others using the blended learning platform (ChaoXing learning system). |  |  |  |  |  |
| 3. I can easily understand the lessons presented using the blended learning platform (ChaoXing learning system). |  |  |  |  |  |
| 4. Clarifying the lessons discussed is easy with the use of the blended learning platform (ChaoXing learning system) |  |  |  |  |  |
| 5. Submitting my assignments using the blended learning platform (ChaoXing learning system) is not complicated. |  |  |  |  |  |
| **Computer Self-Efficacy** | **5** | **4** | **3** | **2** | **1** |
| 1. I feel comfortable using the blended learning platform (ChaoXing learning system). |  |  |  |  |  |
| 2. I am confident that I can easily operate the blended learning platform (ChaoXing learning system) according to my needs. |  |  |  |  |  |
| 3. I believe that I can use the blended learning platform (ChaoXing learning system) independently. |  |  |  |  |  |
| 4. I believe that when I encounter a problem with the blended learning platform (ChaoXing learning system), I can find a solution. |  |  |  |  |  |
| 5. I believe that I can utilize the use of the blended learning platform (ChaoXing learning system). |  |  |  |  |  |
| **Service Quality** | **5** | **4** | **3** | **2** | **1** |
| 1. There are rich types of resources in the blended learning platform (ChaoXing learning system) which can meet my learning needs. |  |  |  |  |  |
| 2. The quality of the resources in the blended learning platform (ChaoXing learning system) is very high. |  |  |  |  |  |
| 3. The other resources in the blended learning platform (ChaoXing learning system) are rich in information, which is conducive to my study and reference. |  |  |  |  |  |
| 4. The function of the blended learning platform (ChaoXing learning system) can meet my learning needs. |  |  |  |  |  |
| 5. The quality of the blended learning platform (ChaoXing learning system) is stable. |  |  |  |  |  |
| **Skills Engagement** | **5** | **4** | **3** | **2** | **1** |
| 1. I use the blended learning platform (ChaoXing learning system) to carry out learning tasks and improve my ability to determine learning goals. |  |  |  |  |  |
| 2. I use the blended learning platform (ChaoXing learning system) to carry out learning tasks, so that my ability to obtain information is improved. |  |  |  |  |  |
| 3. I use the blended learning platform (ChaoXing learning system) to carry out learning tasks and improve my ability to process and use information. |  |  |  |  |  |
| 4. I use the blended learning platform (ChaoXing learning system) to carry out learning tasks, so that my ability to analyse and solve practical problems is improved. |  |  |  |  |  |
| 5. I use the blended learning platform (ChaoXing learning system) to carry out learning tasks and make my thinking more active. |  |  |  |  |  |
| **Emotional Engagement** | **5** | **4** | **3** | **2** | **1** |
| 1. I enjoy using the blended learning platform (ChaoXing learning system) in carrying out learning tasks. |  |  |  |  |  |
| 2. I am proud of what I’ve accomplished using the blended learning platform (ChaoXing learning system). |  |  |  |  |  |
| 3. I am excited to study knowing that I will be using the blended learning platform (ChaoXing learning system). |  |  |  |  |  |
| 4. I feel excited when I am able to carry out my learning tasks because of the blended learning platform (ChaoXing learning system). |  |  |  |  |  |
| 5. I feel comfortable using the blended learning platform (ChaoXing learning system). |  |  |  |  |  |
| **Participation/Interaction** | **5** | **4** | **3** | **2** | **1** |
| 1. Using the blended learning platform (ChaoXing learning system) to carry out learning tasks, my learning methods are diversified. |  |  |  |  |  |
| 2. Using the blended learning platform (ChaoXing learning system) to carry out learning tasks, I will carry out additional learning activities. |  |  |  |  |  |
| 3. Using the blended learning platform (ChaoXing learning system) to carry out learning tasks, I am more active in solving problems. |  |  |  |  |  |
| 4. Using the blended learning platform (ChaoXing learning system) to carry out learning tasks, I will be more punctual when participating in teaching activities. |  |  |  |  |  |
| 5. Using the blended learning platform (ChaoXing learning system) to carry out learning tasks, I am more curious about learning. |  |  |  |  |  |
| **Performance Engagement** | **5** | **4** | **3** | **2** | **1** |
| 1. Using the blended learning platform (ChaoXing learning system) to carry out learning tasks can improve my learning enthusiasm. |  |  |  |  |  |
| 2. When I use the blended learning platform (ChaoXing learning system) to carry out learning tasks, I study harder. |  |  |  |  |  |
| 3. Use the blended learning platform (ChaoXing learning system) to carry out learning tasks, I will verify the knowledge learned. |  |  |  |  |  |
| 4. Using the blended learning platform (ChaoXing learning system) to carry out learning tasks can improve my self-coordination ability. |  |  |  |  |  |
| 5. Using the blended learning platform (ChaoXing learning system) to carry out learning tasks can improve my academic points. |  |  |  |  |  |
